# Effect of Different Preservation Methods on the Microbiological Quality, Physicochemical, and Antioxidant Properties of Red Prickly Pear (*Opuntia streptacantha*) Juice

**DOI:** 10.3390/foods15020282

**Published:** 2026-01-12

**Authors:** Jorge Alberto Jose-Salazar, Diana Maylet Hernández-Martínez, Eliseo Cristiani-Urbina, Liliana Morales-Barrera

**Affiliations:** 1 Departamento de Ingeniería Bioquímica, Escuela Nacional de Ciencias Biológicas, Instituto Politécnico Nacional, Av. Wilfrido Massieu s/n, Unidad Profesional Adolfo López Mateos, Ciudad de México 07738, Mexico; jorge.a.jose.salazar@gmail.com; 2 Departamento de Biofísica, Escuela Nacional de Ciencias Biológicas, Instituto Politécnico Nacional, Prolongación de Carpio y Plan de Ayala s/n, Col. Santo Tomás, Ciudad de México 11340, Mexico; dhernandezmar@ipn.mx

**Keywords:** pasteurization, vacuum-packaged, total phenols, betalains, microbial load

## Abstract

The red prickly pear (*Opuntia streptacantha*) is a fruit that is distinguished by its sensory properties and high content of bioactive compounds. Its rapid spoilage rate significantly impacts its commercialization, underscoring the urgent need for effective preservation methods. This study investigated the effectiveness of various juice preservation techniques—refrigeration, freezing, pasteurization, and vacuum packaging —in maintaining the microbiological quality, physicochemical properties, and antioxidant capacity of red prickly pear juice during storage. The most effective preservation method was found to be freezing the vacuum-packaged and pasteurized juice, referred to as J4. This method adequately maintained key nutritional and physicochemical qualities after 12 months, which was evidenced by a reduction in the microbial growth and the preservation of pH (4.64), acidity (0.74 g citric acid L^−1^), antioxidant activity (2.6–2.9 mmol TE L^−1^), as well as the content of phenols (506 mg GAE L^−1^), betalains (141.2 mg L^−1^), and total sugars (125 g L^−1^). Furthermore, sensory analysis comparing J4-treated juice to control juice revealed no significant differences, confirming that J4 is an effective method for preserving the nutritional, functional, and sensory qualities of red prickly pear juice.

## 1. Introduction

Prickly pear is the fruit of the nopal cactus (*Opuntia* spp.), a group of species native to the Americas and widely distributed in arid and semi-arid regions worldwide. The fruits contain numerous seeds and are covered by a semi-hard peel bearing prominent spines. In Mexico, 23 edible species have been identified, differentiated by their skin color: white, purple, red, orange, and yellow. Prickly pear is primarily consumed fresh and peeled, but it is also widely used in the production of traditional foods such as jams, purées, pulps, juices, and alcoholic beverages [1].

Due to its high moisture content (84–90%) and the presence of readily assimilable sugars such as glucose and fructose (10–15%), prickly pear provides an ideal environment for microbial proliferation [2]. Consequently, its shelf life is limited to just 3 to 4 weeks after harvest [3]. Although Mexico is the world’s leading producer of prickly pear, with an estimated annual yield of approximately 471,000 tons [4], only a small fraction of this production reaches the market. This is primarily due to the lack of effective and affordable preservation methods that enable proper transportation and storage [1]. As a result, prickly pear varieties are typically consumed locally and only during their harvest season [5].

Among the prickly pear species found in Mexico, the red prickly pear stands out due to its high content of bioactive compounds—including polyphenols, betalains, carotenoids, phytosterols, and dietary fiber—which are associated with antioxidant, antiatherogenic, and antiulcerogenic properties [6]. Despite its nutritional potential, it remains underutilized. Extending its shelf life would enable its broader application as a functional food.

Various strategies have been developed to extend the shelf life of fruits while preserving their functional and sensory properties. This is essential because natural metabolic processes—such as respiration and enzymatic reactions—can alter the appearance, texture, and microbiological quality of the fruit [1]. Preservation methods include refrigeration (1 °C to 4 °C), supercooling (–4 °C to 0.5 °C), sub-freezing (−14 °C to –4 °C), freezing (−35 °C to −18 °C), thermal treatments (e.g., pasteurization, scalding, and sterilization), modified atmosphere packaging, cold plasma, pulsed light, ultrasound, ultraviolet light, and ozonated water [2,3].

Refrigeration and freezing are among the most widely used preservation methods due to their relative simplicity, lower cost compared to other techniques, and their ability to retain the nutritional properties, aroma, and flavor of fruits and vegetables [4]. However, freezing requires substantial energy input, and ice crystal formation during the process can disrupt cellular structures. Upon thawing, this disruption may compromise the fruit’s integrity, negatively affecting its quality. Despite these limitations, freezing remains a highly effective method for preserving nutrients, as demonstrated in studies involving various fruits and vegetables, including carrots, cauliflower, garlic, potatoes, blueberries, blackberries, strawberries, and others [4,5,6].

Pasteurization is another thermal preservation method that effectively reduces microbial load and inhibits enzymatic activity [6,7]. It is commonly applied to juices and canned fruits such as oranges, tomatoes, papayas, mangoes, and lychees. While pasteurization can significantly extend the shelf life of food products, it may also cause changes in color, aroma, and texture, and can lead to the degradation of certain nutrients, including vitamins, carotenoids, and phenolic compounds [8]

Vacuum packaging is a more advanced and cost-effective preservation method [2] that creates an environment with low oxygen and elevated carbon dioxide levels. This modified atmosphere slows metabolic and respiratory activity, reduces oxidative processes, delays tissue senescence, decreases ethylene production, decreases the growth of aerobic microorganisms, and inhibits certain enzymatic reactions [9]. This technology has been successfully applied to products such as papaya, beans, and chili peppers, extending their shelf life when combined with refrigeration [10,11].

The combination of multiple fruit preservation methods can offer significant advantages, including extended shelf life, retention of sensory attributes, and economic benefits for regions where these fruits are produced seasonally [1]. However, selecting an appropriate preservation strategy depends on the characteristics of the raw material and the need to minimize adverse effects on its physicochemical, nutritional, and sensory properties. Furthermore, sustainability, the technology’s economic feasibility, and—most importantly—consumer acceptance of the treated product, must be considered [3].

This study investigated the most appropriate method for preserving red prickly pear juice for 12 months (sufficient time for the production of the next harvest) by evaluating the effects of refrigeration, freezing, and the combination of vacuum sealing and pasteurization on bioactive compounds, physicochemical and antioxidant properties, and microbiological safety. Finally, the juice preserved for one year using the selected method was subjected to sensory analysis, which revealed that, in addition to the evaluated qualities, the product was fully accepted by consumers.

## 2. Material and Methods

### 2.1. Raw Materials and Processing

**Figure 1:**
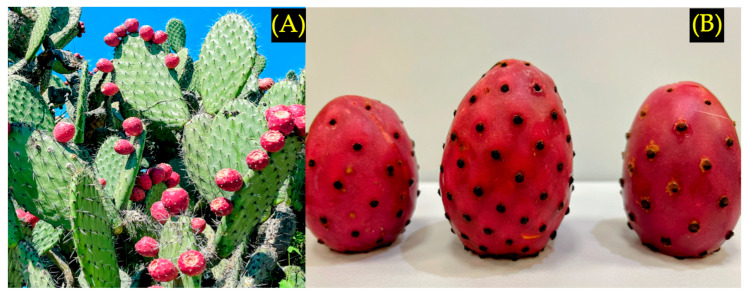
Prickly pear red fruit (**A**) on the nopal plant (*Opuntia streptacantha*) and (**B**) after harvesting.

Red prickly pear (*Opuntia streptacantha*) was collected from nopal plants (Figure 1A) in the locality of El Azafrán, State of Mexico, Mexico (20°02′26.4″ N 99°43′29.0″ W) during September 2024 and 2025.

After harvesting, the fruits were processed individually, removing foreign matter and discarding samples with apparent damage or showing a certain degree of decomposition. Subsequently, the fruits were washed manually with running water, followed by a disinfection process with 1% (*v*/*v*) sodium hypochlorite (Figure 1B). A representative sample of fifty fruits was used to measure length, diameter, weight, and pulp percentage.

### 2.2. Extracting Red Prickly Pear Juice and Preservation

A selection of representative red prickly pear fruits, previously washed and disinfected, was stored intact under refrigeration at 4 °C. From this batch, a portion was manually peeled and deseeded to extract juice containing pulp. A 250 mL sample of freshly harvested fruit juice with pulp (JC) was used to determine its initial physicochemical properties, antioxidant capacity, and microbiological quality. These data were used as a control to determine changes in juice characteristics over time, depending on the preservation method and thermal treatment applied. The remaining intact fruits were kept refrigerated at 4 °C until juice extraction for subsequent analyses (juice extracted from intact fruit stored at 4 °C, J1).

The remaining washed fruits were used to extract juice following the procedure described above. All red prickly pear juice was packed in 250 mL polyethylene bags and vacuum-sealed using a domestic system (FoodSaver^®^ VS3192, Oster, Mexico City, Mexico) at a vacuum pressure of 56 cm Hg. Some sealed bags were stored under refrigeration at 4 °C (vacuum-packaged juice with pulp stored at 4 °C, J2), others were frozen at −20 °C (vacuum-packaged juice with pulp stored at −20 °C, J3), and the remaining bags were pasteurized in their original packaging (90 °C for 30 min, followed by immersion in an ice bath for 15 min) before being stored at −20 °C (vacuum-packaged, pasteurized juice with pulp stored at −20 °C, J4). Treatment J1 was included as a reference condition to evaluate the effect of refrigerated storage of intact fruits prior to juice extraction and was not considered a juice preservation method.

### 2.3. Analysis of Prickly Pear Juice During Storage

To monitor changes in physicochemical properties, antioxidant capacity, and microbiological quality of the juice during storage under each preservation method, samples were evaluated at different times: initially, using JC as the control; after 15 days (0.5 months); and then monthly for a full year after harvest.

The pH was measured using a properly calibrated glass electrode at 20 °C (Oakton OKT35613-54 potentiometer, Cole-Parmer, Vernon Hills, IL, USA). Acidity was determined by titration with 0.1 N NaOH until a pH of 8.1 was reached [12].

For color evaluation, a colorimeter (Colorflex EZ, HunterLab, Reston, VA, USA) was used, and the results were expressed in cylindrical coordinates: a* (red/greenness), b* (yellow/blueness), and L* (lightness/brightness). These values were used to calculate chroma (Equation (1)) and total color difference (TCD, Equation (2)). Based on TCD values, color differences were classified as follows: (1) TCD > 3 = very dissimilar, (2) 1.5 < TCD < 3 = dissimilar, and (3) TCD < 1.5 = small difference. TCD values greater than 3 are generally considered indicative of undesirable color changes in many food products [8].

(1) $$Chroma=\sqrt{a^{*2}+b^{*2}}$$

(2) $$TCD=\sqrt{{\left(∆L^{*}\right)}^{2}+{\left(∆a^{*}\right)}^{2}+{\left(∆b^{*}\right)}^{2}}$$

where ΔL*, Δa*, and Δb* represent the differences in color parameters between the control sample (JC) and the treated samples (J1, J2, J3, and J4) throughout the storage period.

The total sugar concentration was quantified using the sulfuric acid–UV method [13]. Total phenol content was determined using the 10% Folin–Ciocalteu reagent [14]. Betalain content was estimated using a spectrophotometric method after diluting the samples (1:20) [15]. Antioxidant capacity was expressed in mg L^−1^ Trolox Equivalents (TE) and evaluated using the 2,2′-azino-bis(3-ethylbenzothiazoline-6-sulfonic acid) (ABTS) radical (Sigma Aldrich, Toluca, Mexico State, Mexico) [14], the 2,2-diphenyl-1-picrylhydrazyl (DPPH) radical (Sigma Aldrich, Toluca, Mexico State, Mexico) [16], and the Ferric Reducing Antioxidant Power (FRAP) assay using 2,4,6-tris(2-pyridyl)-(S)-triazine (TPTZ) (Sigma Aldrich, Toluca, Mexico State, Mexico) [17]. Microbiological quality was assessed by verifying and quantifying the presence of total coliforms [18], aerobic mesophiles [19], filamentous fungi, and yeasts [20], expressed as colony-forming units (CFUs). Fresh juice (JC) was used as the microbiological reference to represent the baseline microbial status prior to the application of any preservation treatment, enabling direct comparison among preservation strategies.

### 2.4. Sensory Analysis of Juice

A group of 30 participants conducted a sensory evaluation of JC and J4 after 1 year of storage. Each panelist received a 15 mL sample of each juice, served at 5 °C, and evaluated the following attributes: color, odor, flavor, acidity, sweetness, texture, astringency, aftertaste, and overall acceptance. A five-point hedonic scale was used, where 5 indicated “like it very much,” 4 “like it moderately,” 3 “neither like nor dislike,” 2 “dislike it moderately,” and 1 “dislike it very much.” Finally, an affective paired-preference test was conducted, in which participants indicated their preference for either JC or J4.

### 2.5. Statistical Analysis

The results presented are the average of at least three replicates for physicochemical analyses, antioxidant capacity, and microbiological quality. A one-way analysis of variance (ANOVA), followed by Dunnett’s test and Student’s *t*-test, was performed to identify statistically significant differences among data sets (*p* < 0.05), using GraphPad Prism software version 10.0 (GraphPad Software, La Jolla, CA, USA).

Chi-square (X^2^) statistical analysis was applied to the sensory evaluation data, using the minimum number of acceptable responses required to reach statistical significance [21].

## 3. Results and Discussion

### 3.1. Physical Characteristics of Intact Red Prickly Pear

The results obtained for the weight, diameter, length, and pulp content of red prickly pear used in this study are presented in Table 1. Both the weight and pulp content were lower than those reported by previous studies. These differences may be attributed to various factors, including the specific variety of prickly pear and environmental conditions—such as climate, rainfall, geographic region, daylight duration, and harvest season—which significantly influence the physical characteristics of *Opuntia streptacantha* fruits [22,23,24].

### 3.2. Microbial Load

The fruit of *Opuntia streptacantha* has a high sugar content and low acidity, making it highly susceptible to microbial growth and significantly reducing its shelf life as a fresh product [2]. Therefore, microbiological analyses were conducted to monitor the juice during storage and identify the most effective preservation method to maintain microbiological quality over 1 year, ensuring consumer safety. Table 2 presents the results for aerobic mesophiles (AM), total coliform bacteria (TCB), filamentous fungi (F), and yeasts (Y) under each preservation condition.

The JC sample did not contain total coliform bacteria (TCB) or filamentous fungi (F). However, aerobic mesophiles (AM) and yeasts (Y) were detected, with counts of 2.0 × 10^4^ CFU mL^−1^ and 1300 CFU mL^−1^, respectively. The microbiological profile of J1 after two weeks of refrigerated storage did not differ significantly from that of JC (TCB: 0, AM: 2.1 × 10^4^ CFU mL^−1^, F: 0, Y: 1100 CFU mL^−1^), indicating that the fruit peel effectively preserves microbiological quality under short-term refrigeration.

Rodrigues et al. [29] reported that red prickly pear can be preserved for up to six weeks using edible coatings, such as alginate and chitosan, in combination with refrigeration, resulting in lower microbial contamination. In contrast, uncoated samples deteriorated after just two weeks of refrigerated storage, suggesting that the peel alone is insufficient for long-term preservation.

**Figure 2:**
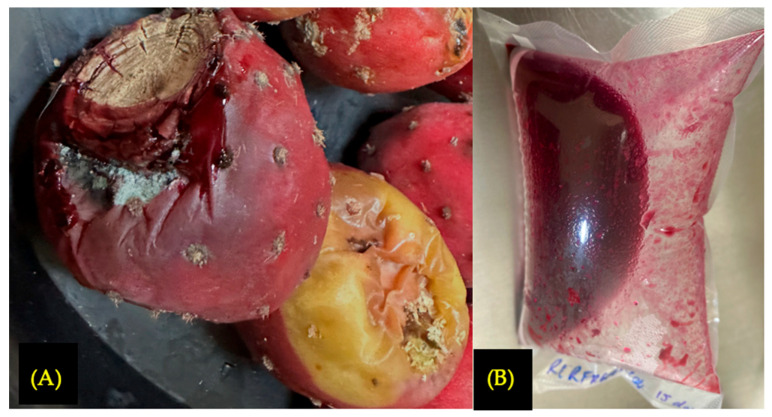
Intact fruit refrigerated at 4 °C after 21 days of storage (**A**); vacuum-packaged and refrigerated juice with pulp (J2) after 1 month of storage (**B**).

Shortly before conducting the evaluation corresponding to the first month of storage of the intact fruit, visible microbial growth was observed on the surface of the fruit skin (Figure 2A). As a result, the sample could not be preserved under refrigerated conditions for further analysis beyond 15 days after harvest.

In the case of J2, no fungal growth (F = 0 CFU mL^−1^) was detected during the first month of storage. However, after this period, a significant increase in other microorganisms was observed compared to the levels recorded 15 days after harvest: (AM) increased from 5 × 10^4^ to 4.3 × 10^6^ CFU mL^−1^, (Y) from 1300 to 1500 CFU mL^−1^, and (TCB) from 500 to 700 CFU mL^−1^. The vacuum-sealed bags expanded due to CO_2_ production (Figure 2B), indicating intense microbial activity. Consequently, the samples were deemed unsuitable for consumption and were discarded.

For J3, no fungal growth was observed at any point during the study. It is important to note that TCB was quantified after 15 days of storage for both J2 and J3, whereas it was not detected in the control sample. This may be attributed to handling during processing [30,31]

During the first four months of J3 storage, AM counts ranged from 1 × 10^4^ to 5 × 10^4^ CFU mL^−1^. In the fifth month, a logarithmic increase was observed (from 2.4 × 10^4^ to 2.6 × 10^5^ CFU mL^−1^), followed by another significant rise in the sixth month (up to 9.6 × 10^5^ CFU mL^−1^). Additionally, yeast counts decreased from 1300 CFU mL^−1^ in JC to 310 CFU mL^−1^ in J3, suggesting that certain yeast strains may be sensitive to low temperatures (−20 °C), resulting in reduced viability [32]. These findings indicate that this preservation method is effective for up to four months.

The microbiological results for J2 and J3, compared with JC and J1, revealed differences in the presence of aerobic mesophiles and total coliform bacteria. These differences may be attributed to microorganisms associated with the fruit peel that were present at very low levels and therefore not detected at time zero. The thawing of frozen samples prior to analysis, along with freeze-induced structural damage, may promote the release of intracellular nutrients and the recovery of sublethally injured microorganisms, thereby explaining the observed values [33,34,35]. Such effects are commonly reported in frozen food systems and are associated with handling and processing steps rather than microbial growth during frozen storage [36].

In contrast, no microbial growth was detected in J4 throughout the 12-month storage period. Even the AM and yeasts present in JC were eliminated. Ferreira et al. [6] reported a reduction in microbial load in red prickly pear samples subjected to thermal pasteurization at 71.1 °C for 30 s; however, this method only extended shelf life by 22 days, as microbial counts remained high (>1 × 10^6^ CFU mL^−1^). In the present study, a complete reduction in microorganisms (from 2 × 10^4^ to 0 CFU mL^−1^) was achieved using pasteurization at 90 °C for 30 min, followed by freezing, demonstrating the superior effectiveness of this method for long-term preservation.

### 3.3. Physical and Chemical Characteristics of Red Prickly Pear

#### 3.3.1. pH and Titratable Acidity

**Figure 3:**
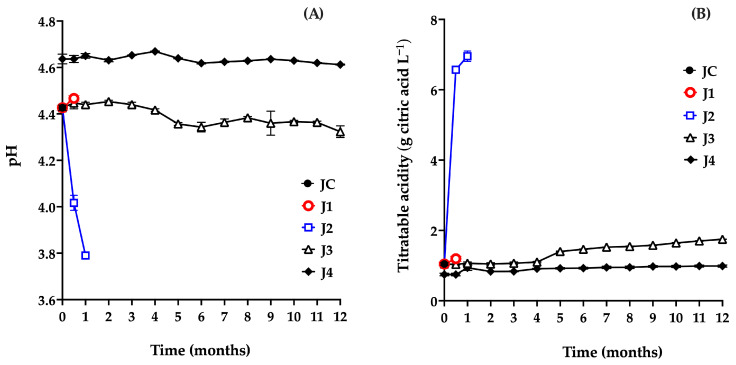
Evaluation of pH (**A**) and titratable acidity (**B**) of red prickly pear juice during storage. Freshly harvested fruit juice with pulp (JC); juice extracted from intact fruit stored at 4 °C (J1); vacuum-packaged juice with pulp stored at 4 °C (J2); vacuum-packaged juice with pulp stored at −20 °C (J3); and vacuum-packaged, pasteurized juice with pulp stored at −20 °C (J4). Error bars are present but may not be visible when smaller than the symbol size.

Both pH and titratable acidity are key indicators in fruit preservation, as they directly influence the product’s sensory characteristics. Variations in these parameters are closely associated with microbial activity and can therefore serve as reliable markers for determining whether a product remains suitable for consumption [37]. The results for the juice samples’ pH (Figure 3A) and titratable acidity (Figure 3B) are shown below.

The pH value of JC (4.43) was within the previously reported range for this fruit (4.5–6.38), confirming its acidic nature. This parameter is a key physiological trait, closely linked to shelf life and fruit quality. Moreover, pH determination helps assess whether the juice is suitable for use in the production of various food products. In this context, values equal to or below 4 may indicate the onset of decomposition, compromising both the safety and the technological viability of the fruit [25].

In J1, 15 days post-harvest, no significant differences were observed in pH (4.46; *p* = 0.6667) or titratable acidity (1.19 g citric acid L^−1^; *p* = 0.6667) compared to the control sample (JC: pH 4.43, acidity 1.045 g citric acid L^−1^) (Figure 3A,B). However, further monitoring of J1 was not possible due to deterioration of the fruit caused by microbial growth, as shown in Figure 2A.

J2 exhibited a marked decrease in pH (from 4.42 to 3.79) and a significant increase in titratable acidity (from 1.045 to 6.95 g citric acid L^−1^) during the first month of storage (Figure 3A,B). Figure 2B shows gas production associated with bag bulging, indicating spoilage. These changes are consistent with microbiological findings, particularly the increase in AM, which acidifies the medium by producing organic acids such as lactic acid [37,38]. Based on these results, refrigeration alone is not considered a suitable method for preserving the juice.

Similarly, J3 showed a significant decrease in pH (*p* ≤ 0.0001) after 5 months (4.43 to 4.35), along with a significant increase in titratable acidity (*p* ≤ 0.0001) from 1.045 to 1.40 g citric acid L^−1^, attributed to microbial growth. These findings confirm that this preservation method is ineffective beyond four months.

In contrast, J4 showed an increase in pH from 4.43 (JC) to 4.64, and a decrease in acidity from 1.04 to 0.74 g citric acid L^−1^, due to pasteurization at the beginning of storage (t = 0 months). Pasteurization induces chemical reactions that alter the physicochemical, nutritional, and sensory properties of food products [39]. Several studies have reported increases in pH following pasteurization in juices from fruits, such as carambola (2.4 to 2.71) [40], kutkura (3.61 to 3.74) [39], and *Morinda citrifolia* (3.86 to 4.06) [41]. This increase in pH is commonly associated with a reduction in titratable acidity due to the degradation of organic acids during thermal processing. Heat promotes oxidation, decarboxylation, and other thermally induced reactions that lead to the decomposition of organic acids. Ascorbic acid, for instance, undergoes accelerated oxidation when exposed to heat, resulting in the formation of dehydroascorbic acid and subsequent degradation products, which alter proton availability and reduce the overall acidity of the medium [42,43,44,45].

The initial pH and acidity values of J4 (4.64 and 0.9 g citric acid L^−1^, respectively) are consistent with those reported for various red prickly pear cultivars [25,46]. A decrease in pH and an increase in acidity during storage would suggest microbial activity and spoilage. However, in this study, the J4 treatment maintained values close to those of fresh juice throughout the 12 months, indicating that the method effectively preserved the product without compromising its quality.

#### 3.3.2. Variation in Total Sugar Content

**Figure 4:**
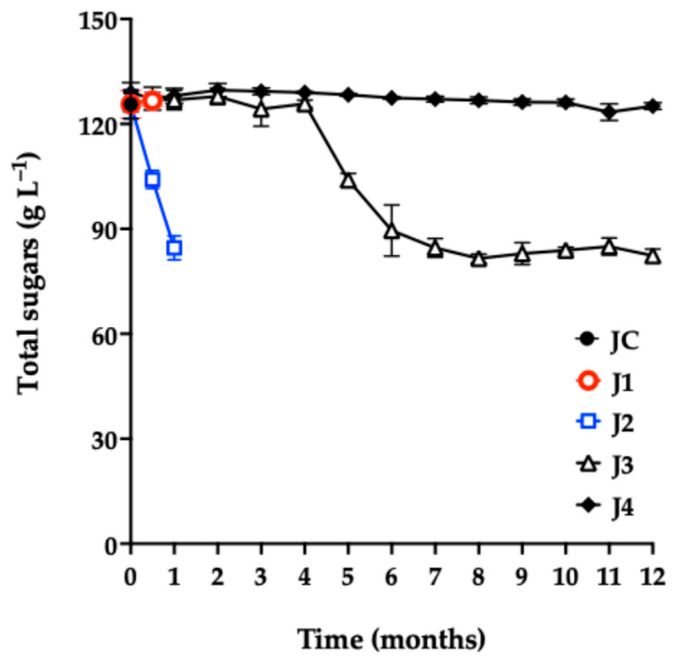
Total sugar content of red prickly pear juice during storage. Freshly harvested fruit juice with pulp (JC); juice extracted from intact fruit stored at 4 °C (J1); vacuum-packaged juice with pulp stored at 4 °C (J2); vacuum-packaged juice with pulp stored at −20 °C (J3); and vacuum-packaged, pasteurized juice with pulp stored at −20 °C (J4). Error bars are present but may not be visible when smaller than the symbol size.

The total sugar content in JC (Figure 4) was 125 g L^−1^. Other studies report values between 50 and 164 g L^−1^ [3,6,47,48].

As shown in Figure 4, there was no significant difference (*p* = 0.4719) in total sugar content between J1 and JC at 0.5 months of storage. In contrast, J2 showed a significant decrease in sugar concentration compared with the control, consistent with the observed microbial growth in this sample.

During the first four months of storage, J3 showed no significant differences in sugar content compared to JC. However, in the fifth month, a noticeable reduction was observed, attributed to the proliferation of aerobic mesophiles, as confirmed by microbiological quality analyses.

Notably, J4 maintained stable sugar content throughout the 12-month storage period, with no significant differences compared to JC (*p* = 0.3567). This result aligns with the excellent microbiological quality observed in J4, confirming the preservation method’s effectiveness in maintaining the nutritional integrity of the juice.

### 3.4. Variation in Total Phenol and Betalain Content

**Figure 5:**
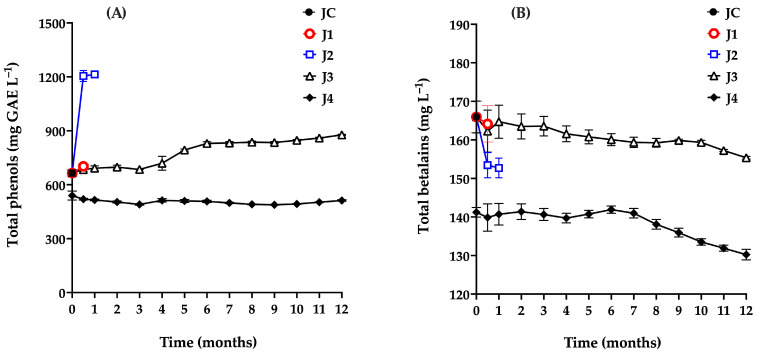
Total phenol (**A**) and betalains (**B**) content of red prickly pear juice during storage. Freshly harvested fruit juice with pulp (JC); juice extracted from intact fruit stored at 4 °C (J1); vacuum-packaged juice with pulp stored at 4 °C (J2); vacuum-packaged juice with pulp stored at −20 °C (J3); and vacuum-packaged, pasteurized juice with pulp stored at −20 °C (J4). Error bars are present but may not be visible when smaller than the symbol size.

The monitoring of total phenol content in samples treated and stored using different preservation methods is shown in Figure 5A. The total phenol content in the control sample (JC) was 665.22 mg GAE L^−1^, which is consistent with values reported in red prickly pear from the Zacatecas region (670 mg GAE L^−1^), and notably higher than those found in white prickly pear (242 mg GAE L^−1^), and yellow prickly pear (247 mg GAE L^−1^). Compared to other fruits, red prickly pear has a lower phenolic content than blueberries (1364.52 mg GAE L^−1^) and purple grapes (1117.10 mg GAE L^−1^), but higher than pomegranate (138 mg GAE L^−1^) [5,49,50,51,52].

Additionally, betalains were monitored (Figure 5B). In the control sample (JC), a concentration of 165.95 mg L^−1^ was recorded, which is similar to the 120 mg L^−1^ reported by other authors in red prickly pear [5,53]. This fruit exhibits lower betalain concentrations than those found in beet juice (800–1300 mg L^−1^) [54] and blood berry juice (up to 3600 mg L^−1^) [55]. In contrast, garambullo fruit, another cactus family fruit, contains significantly less betalains (75.8 mg L^−1^) [56] than the red prickly pear analyzed in this study.

Betalains are natural pigments widely distributed in plants. They are responsible for the characteristic yellow hues (betaxanthins) and red-violet hues (betacyanins) observed in flowers and fruits [57]. These compounds possess phenolic groups and cyclic amines, which confer potent free radical scavenging activity. This property has promoted their use in the food industry as natural colorants with both functional and nutritional value [58].

Sample J1 (t = 0.5 months) did not show significant changes (*p* > 0.05) in total phenol or betalains content (702 mg L^−1^ and 164.15 mg L^−1^, respectively) compared to JC.

Sample J2 exhibited an 82.6% increase in total phenol content after 15 days of storage. Li et al. [59], Tkacz et al. [54], and Kwaw et al. [60] attributed similar phenomena in blueberry, apple, and mulberry juices, respectively, to enzymatic activity from certain mesophile microorganisms, particularly lactic acid bacteria. These microorganisms are known to hydrolyze glycosylated phenolic compounds via enzymatic activity (e.g., β-glucosidase), releasing simpler phenolic groups from plant cell walls that are more readily quantified, thereby increasing the measured total phenol content. Conversely, J2 showed a slight decrease in betalain content, from 165 to 152 mg L^−1^, a trend previously reported in studies suggesting that betalains are chemically less stable and may degrade or be metabolized by specific microbial populations, particularly lactic acid bacteria, as observed in lactic-fermented vegetable juices. The decrease in betalain content may also be associated with the hydrolysis of betanin (betanidin-5-O-β-glucoside), whereby cleavage of the β-glycosidic bond yields glucose and the aglycone betanidin. The resulting aglycone exhibits lower chemical stability and may undergo further degradation through oxidative or pH-dependent reactions, contributing to the observed reduction in total betalain content [56].

A qualitatively similar behavior was observed for sample J3, in which total phenol content increased significantly over time, while betalain levels declined. As mentioned above, this trend has been correlated with the proliferation of aerobic mesophiles, particularly lactic acid bacteria. Based on these findings, the maximum recommended storage time for J3 under the tested conditions is four months. 

Initially, J4 showed a reduction in both total phenols (506 mg GAE L^−1^) and betalains (141.2 mg L^−1^) due to heat treatment, compared to JC (665 mg GAE L^−1^ and 165.95 mg L^−1^). Throughout the 12-month storage period, total phenol levels remained nearly constant, while betalain content began to decline after the seventh month. From months 7 to 12, an overall decrease of approximately 7.62% was observed; however, this reduction was not statistically significant (*p* = 0.4846). Ferreira et al. [61] similarly observed betalain degradation over a 40-day storage period, attributing it to betalains’ sensitivity to factors such as pH, oxygen exposure, metal ions, storage temperature, water activity, light, and enzymatic reactions, all of which accelerate decomposition [62].

In this context, J4 maintains betalain levels above 130 mg L^−1^ after 12 months—compared to the reported range of 60–130 mg L^−1^ in red prickly pear [5,53,61]—which is noteworthy and confirms the effectiveness of this preservation method in maintaining the integrity of the juice.

### 3.5. Variation in the Antioxidant Capacity of Juices

Due to the diverse nature of the antioxidant compounds present in the fruit, multiple methods were employed to evaluate its antioxidant capacity—namely, the ABTS, DPPH, and FRAP assays. These techniques address different mechanisms of action: FRAP measures electron transfer, while ABTS and DPPH assess hydrogen atom donation [63,64]. The combined use of these assays enables a more comprehensive and representative characterization of the fruit’s antioxidant capacity, particularly in complex matrices such as fruits, which contain a wide array of bioactive compounds involved in various redox reactions [65].

**Figure 6:**
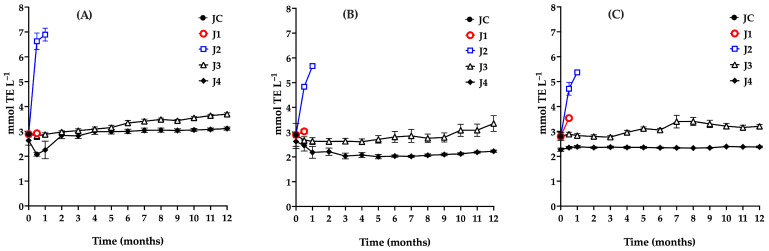
Evaluation of the antioxidant capacity during storage of red prickly pear juice using the ABTS (**A**), DPPH (**B**), and FRAP (**C**) assays. Freshly harvested fruit juice with pulp (JC); juice extracted from intact fruit stored at 4 °C (J1); vacuum-packaged juice with pulp stored at 4 °C (J2); vacuum-packaged juice with pulp stored at −20 °C (J3); and vacuum-packaged, pasteurized juice with pulp stored at −20 °C (J4). Error bars are present but may not be visible when smaller than the symbol size.

Accordingly, the antioxidant capacity of the stored samples was monitored using the ABTS, FRAP, and DPPH assays (Figure 6A–C). The results are presented below.

The antioxidant capacity results from the three JC assays showed no significant differences (*p* > 0.05), with a value of 2.9 mmol TE L^−1^. These findings are consistent with those reported by Sumaya et al. [66], who observed antioxidant capacities ranging from 2 to 12 mmol TE L^−1^ in red prickly pear juice using the DPPH assay. Sample J1 maintained a stable antioxidant capacity during the 15-day storage period, with no significant changes observed in ABTS (*p* = 0.4232), DPPH (*p* = 0.6667), or FRAP (*p* = 0.6667) under the specified conditions.

Sample J2 exhibited a substantial increase in antioxidant capacity between days 15 and 30 of storage across all three assays, with ABTS values rising from 2.88 to 6.89 mmol TE L^−1^. This increase is attributed to the proliferation of lactic acid bacteria, which induced various changes in juice preservation, including alterations in pH, titratable acidity, an increase in total phenolic content due to microbial hydrolysis of phenolic conjugates, a decrease in betalains as a result of their degradation or microbial utilization, and consequent changes in antioxidant capacity. Li et al. [59] noted that increases in total phenol content and antioxidant capacity are associated with shifts in phenolic profiles. In J2, a marked rise in total phenol concentration during storage contributed to the enhanced antioxidant capacity.

In the case of J3, a significant increase in antioxidant capacity was observed from the fifth month onward, as indicated by the ABTS assay (*p* ≤ 0.0001), rising from 2.9 to 3.69 mmol TE L^−1^. This trend reflects changes in physicochemical and phytochemical parameters, driven by microbial proliferation after the fourth month. Consequently, the recommended maximum storage duration for J3 under the tested conditions is four months.

During storage, a gradual decline in betalain content was observed in sample J4, attributable to the thermal treatment applied prior to storage, whereas total phenolic content remained nearly constant. Given betalains’ key role in the antioxidant properties of prickly pear juice, this compositional change helps explain the initial reduction in antioxidant capacity observed after pasteurization.

At time zero, pasteurization resulted in a 10% decrease in antioxidant capacity as measured by the ABTS and DPPH assays (from 2.9 to 2.6 mmol TE L^−1^). In contrast, a more pronounced reduction (22%) was detected using the FRAP assay. This difference is associated with partial degradation of betalains during thermal treatment, which directly affects the antioxidant capacity quantified by the FRAP assay.

Despite a decline in betalains and stability in total phenolic content, antioxidant capacity, as measured by the ABTS assay, increased during storage. This behavior can be attributed to the enhanced accessibility and transformation of hydrophilic antioxidant compounds during processing and storage, including free phenols, polyphenols, betalains, water-soluble flavonoids, low-molecular-weight phenolic derivatives, degradation products, and early-stage Maillard reaction products [67,68].

The ABTS assay, which involves both single-electron transfer (SET) and hydrogen-atom transfer (HAT) mechanisms and employs a water-soluble radical cation, is particularly sensitive to these compounds. In contrast, DPPH may underestimate antioxidant activity due to its limited solubility in aqueous systems, and FRAP, conducted under acidic conditions, may not fully capture the antioxidant activity of certain phenolic derivatives [67]. Together, these methodological differences explain the observed increase in ABTS antioxidant capacity during storage despite relatively stable total phenolic levels [42,43,59,67,68,69,70].

### 3.6. Color Variation

The color of fruit juice can be affected by various factors, including improper storage, which may lead to microbial proliferation and subsequent alterations in pH and acidity. Additionally, non-enzymatic reactions and thermal processing—commonly applied to extend shelf life—can significantly impact the sensory attributes of the juice, such as color, aroma, and flavor [71,72].

Table 3 presents the color parameters L*, a*, and b*, as well as chroma (C*) and total color difference (TCD) for the different preservation treatments applied to red prickly pear juice. At 0.5 months of storage, the sample J1 showed no perceptible color change compared to the freshly extracted juice (JC), as indicated by a TCD value of 0.49, which is classified as a non-perceptible difference.

On the other hand, sample J2 exhibited, after one month of storage, a significant color difference, with a TCD value of 12.674 compared to JC. This was accompanied by increases in L*, a*, and b* values. These changes are attributed to the stabilization of betacyanins at pH values below 4, which helps preserve the juice’s reddish hue. A similar trend of increased L*, a*, and b* values has been reported by Wang et al. [73] and Değirmencioğlu et al. [74] following bacterial fermentation processes.

In sample J3, the total color difference (TCD) during the first four months of storage was classified as slightly noticeable (<0.7) compared to JC. However, this changed after the fifth month, when the TCD increased from 1.4 to 4.05, indicating a noticeable color change. Based on this observation, along with the physicochemical and antioxidant parameters evaluated, it is concluded that this preservation method is effective for up to four months.

Regarding sample J4, the TCD values suggest that the color was affected by both pasteurization and storage duration, with an average TCD of 2.09, which is considered a noticeable change compared to JC at time zero. Ferreira et al. [3] reported that thermal treatments such as pasteurization accelerate the degradation of betalains. However, degraded betalains may form new pigment compounds that help retain the red coloration, as observed in J4. Additional factors contributing to color changes include non-enzymatic reactions. Medeni et al. [75] noted that thermal processes can degrade pigments such as carotenoids and chlorophyll. However, in this case, the treatment was deemed appropriate because it did not result in undesirable effects, such as darkening due to Maillard reactions.

### 3.7. Sensory Analysis

Red prickly pear juice was evaluated by comparing the freshly extracted sample (JC) with the J4 sample stored for 12 months. The objective was to determine whether the J4 sample remained acceptable to consumers, accounting for the potential effects of pasteurization and long-term storage.

**Figure 7:**
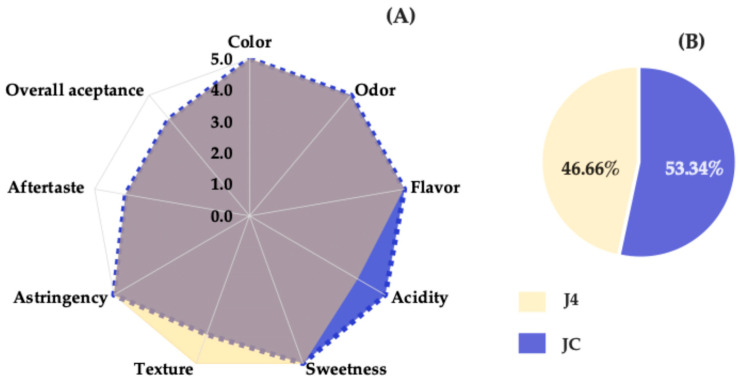
Sensory profile (**A**) and preference test (**B**) for JC and J4. The color overlap in the chart reflects similar values for several sensory attributes, except for acidity and texture, which showed variation between JC and J4.

Sensory evaluation was conducted using a five-point hedonic scale, revealing the highest-rated attributes for each sample. As shown in Figure 7A, the J4 sample received a score of 5.0 for attributes such as color, aroma, flavor, texture, sweetness, and astringency, indicating a pleasant sensory experience and a well-balanced sweetness profile. The attribute aftertaste received a score of 4.0. In contrast, the JC sample was most highly rated for acidity, scoring 5.0, likely due to the presence of betalains and organic acids that contribute to this sensory characteristic [53].

Regarding texture, J4 was rated higher than JC, possibly because the pasteurization process produced a smoother, more fluid consistency. A notable difference between the two samples was observed in the acidity attribute, with J4 exhibiting lower perceived acidity, likely due to reduced organic acids resulting from thermal treatment [39].

Figure 7B shows the results of the preference test, in which 53.34% of the panelists (16 individuals) preferred the fresh juice (JC), while 46.66% (14 individuals) preferred the J4 sample. This difference was not statistically significant, as a minimum of 21 responses favoring one sample would be required to establish a significant preference according to the pairwise preference test tables (two-tailed, *p* = ½) for a panel of 30 judges [21]. Therefore, no significant preference or rejection was detected between JC and J4. It can be concluded that the preservation method applied to J4 was effective in maintaining the sensory quality and consumer acceptance of red prickly pear juice over a 12-month storage period.

## 4. Conclusions

After 12 months of storage, the most effective method for preserving red prickly pear juice was treatment J4. This approach resulted in no detectable growth of total coliforms, aerobic mesophiles, filamentous fungi, or yeasts. The physicochemical parameters remained stable, with an average pH of 4.635, titratable acidity of 0.9059 g citric acid L^−1^, total phenols of 506 mg GAE L^−1^, betalains of 138.4 mg L^−1^, and total sugars of 127.3 g L^−1^. Antioxidant capacity was also preserved, with values of 2.977 mmol TE L^−1^ (ABTS), 2.169 mmol TE L^−1^ (DPPH), and 2.353 mmol TE L^−1^ (FRAP), comparable to those of freshly extracted juice (JC). This preservation method effectively maintained the physicochemical properties, antioxidant capacity, microbiological safety, and sensory quality of the juice throughout the 12-month storage period. Moreover, it offers a cost-effective and accessible alternative to more complex techniques such as freeze-drying or spray drying, yielding a product ready for consumption and suitable as a raw material for further food processing applications. Future studies should evaluate emerging non-thermal physical preservation technologies, such as ultraviolet radiation or ultrasound, to further enhance the quality and shelf-life of prickly pear juice.

## Figures and Tables

**Table 1:** Physical characteristics of red prickly pear fruit.

Characteristic	Result	Reported Range
Weight (g)	48.95 ± 10.31	60–64 [23,24,25]
Length (cm)	4.56 ± 0.48	4.4–5.12 [25]
Diameter (cm)	4.13 ± 0.34	4.06–4.42 [25]
Pulp content (%)	38.75 ± 0.78	43–57 [26]

*n* = 50. values are expressed as mean ± SD.

**Table 2:** Microbial load during storage.

Juice	Time (Months)	TCB (CFUs mL^−1^)	AM (CFUs mL^−1^)	Y (CFUs mL^−1^)	F (CFUs mL^−1^)
JC	0	0 ^a^	2 × 10^4^ ± 1 × 10^3 a^	1300 ± 173 ^a^	0
J1	0.5	0 ^a^	2.1 × 10^4^ ± 1.5 × 10^3^ *	1100 ± 265 ^a^	0
J2	0.5	500 ± 80 *	5 × 10^4^ ± 2 × 10^3 a^	1300 ± 200 ^a^	0
	1	700 ± 20 *	4.3 × 10^6^ ± 2.3 × 10^5^ *	1500 ± 200 ^a^	0
J3	0.5	110 ± 20 *	2.8 × 10^4^ ± 2 × 10^3 a^	310 ± 36 *	0
	1	170 ± 10 *	5 × 10^4^ ± 1 × 10^3 a^	260 ± 50 *	0
	2	100 ± 20 *	4.9 × 10^4^ ± 1 × 10^3 a^	400 ± 30 *	0
	3	110 ± 10 *	1 × 10^4^ ± 1 × 10^3 a^	290 ± 26 *	0
	4	83 ± 6 *	2.4 × 10^4^ ± 4 × 10^3 a^	300 ± 20 *	0
	5	106 ± 5 *	2.6 × 10^5^ ± 2 × 10^4^ *	250 ± 5 *	0
	6	50 ± 5 *	9.6 × 10^5^ ± 2 × 10^4^ *	260 ± 13 *	0
	7	20 ± 5 ^a^	5.8 × 10^5^ ± 1.2 × 10^4^ *	210 ± 10 *	0
	8	30 ± 5 *	2.9 × 10^5^ ± 1 × 10^4^ *	300 ± 26 *	0
	9	35 ± 5 *	2.3 × 10^5^ ± 3.6 × 10^4^ *	210 ± 10 *	0
	10	70 ± 5 *	3.2 × 10^5^ ± 3 × 10^4^ *	230 ± 53 *	0
	11	100 ± 10 *	4.1 × 10^5^ ± 1.1 × 10^4^ *	120 ± 36 *	0
	12	0 ^a^	2.9 × 10^5^ ± 1 × 10^4^ *	25 ± 5 *	0
J4	0	0	0	0	0
	0.5	0	0	0	0
	1	0	0	0	0
	2	0	0	0	0
	3	0	0	0	0
	4	0	0	0	0
	5	0	0	0	0
	6	0	0	0	0
	7	0	0	0	0
	8	0	0	0	0
	9	0	0	0	0
	10	0	0	0	0
	11	0	0	0	0
	12	0	0	0	0

*n* = 3; values are expressed as mean ± SD. Freshly harvested fruit juice with pulp (JC); juice extracted from intact fruit stored at 4 °C (J1); vacuum-packaged juice with pulp stored at 4 °C (J2); vacuum-packaged juice with pulp stored at −20 °C (J3); and vacuum-packaged, pasteurized juice with pulp stored at −20 °C (J4). Statistical analysis was performed using one-way ANOVA followed by Dunnett’s test and Student’s *t*-test, as described by Zar et al. [27] and Saneii and Doosti. [28]. Values sharing the same letter as the control (JC) do not differ significantly, whereas values marked with an asterisk indicate statistically significant differences (*p* < 0.05).

**Table 3:** Color variation during storage.

	Time (Months)	L* a* b*	C*	TCD
JC	0	L* = 4.49 ± 0.040 ^a^, a* = 26.620 ± 0.191 ^a^, b* = 6.710 ± 0.141 ^a^	27.453 ± 0.19 ^a^	-
J1	0.5	L* = 4.317 ± 0.174 ^a^, a* = 28.527 ± 0.33 ^a^, b* = 6.140 ± 0.115 *	29.546 ± 0.279 *	0.499 ± 0.320
J2	0.5	L* = 6.380 ± 0.223 *, a* = 33.200 ± 0.220 *, b* = 10.590 ± 0.185 *	35.204 ± 0.246 *	8.607 ± 3.426
	1	L* = 7.593 ± 0.399 *, a* = 39.353 ± 0.133 *, b* = 11.653 ± 0.405 *	41.603 ± 0.325 *	12.674 ± 0.120
J3	0.5	L* = 4.200 ± 0.089 ^a^, a* = 27.077 ± 0.735 ^a^, b* = 7.253 ± 0.153 ^a^	28.031 ± 0.750 ^a^	0.808 ± 0.735
	1	L* = 4.577 ± 0.371 ^a^, a* = 27.670 ± 0.560 ^a^, b* = 6.723 ± 0.239 ^a^	28.476 ± 0.570 ^a^	1.184 ± 0.594
	2	L* = 4.507 ± 0.201 ^a^, a* = 26.850 ± 0.503 ^a^, b* = 7.053 ± 0.205 ^a^	27.761 ± 0.540 ^a^	0.563 ± 0.467
	3	L* = 4.573 ± 0.120 ^a^, a* = 26.877 ± 0.422 ^a^, b* = 7.337 ± 0.189 ^a^	27.861 ± 0.360 ^a^	0.874 ± 0.076
	4	L* = 4.59 ± 0.176 ^a^, a* = 26.740 ± 0.487 ^a^, b* = 7.180 ± 0.160 ^a^	27.688 ± 0.470 ^a^	0.660 ± 0.399
	5	L* = 4.557 ± 0.040 *, a* = 27.657 ± 0.211 ^a^, b* = 7.663 ± 0.184 ^a^	28.699 ± 0.230 ^a^	1.435 ± 0.371
	6	L* = 5.550 ± 0.234 *, a* = 27.843 ± 1.130 ^a^, b* = 6.047 ± 0.277 ^a^	28.493 ± 1.120 ^a^	1.980 ± 0.690
	7	L* = 5.713 ± 0.263 ^a^, a* = 29.390 ± 0.609 ^a^, b* = 6.953 ± 0.457 ^a^	30.202 ± 0.690 ^a^	3.094 ± 0.622
	8	L* = 4.677 ± 0.185 ^a^, a* = 27.983 ± 0.699 ^a^, b* = 7.207 ± 0.357 ^a^	28.897 ± 0.760 ^a^	1.467 ± 0.865
	9	L* = 4.730 ± 0.192 ^a^, a* = 27.780 ± 0.745 ^a^, b* = 6.140 ± 0.397 ^a^	28.451 ± 0.810 ^a^	1.479 ± 0.603
	10	L* = 4.343 ± 0.021 ^a^, a* = 26.347 ± 0.067 ^a^, b* = 6.580 ± 0.218 ^a^	27.157 ± 0.002 ^a^	0.470 ± 0.100
	11	L* = 5.237 ± 0.906 ^a^, a* = 29.483 ± 3.268 ^a^, b* = 8.603 ± 1.596 *	30.717 ± 3.580 *	4.049 ± 2.776
	12	L* = 4.683 ± 0.179 *, a* = 27.537 ± 1.153 ^a^, b* = 6.770 ± 0.295 ^a^	28.36 ± 1.110 ^a^	1.234 ± 0.920
J4	0	L* = 8.553 ± 0.240 *, a* = 35.370 ± 0.128 *, b* = 10.813 ± 0.217 *	36.986 ± 0.110 *	-
	0.5	L* = 8.757 ± 0.165 *, a* = 35.590 ± 0.512 *, b* = 10.333 ± 0.096 *	37.060 ± 0.518 *	0.708 ± 0.280
	1	L* = 8.817 ± 0.100 *, a* = 35.537 ± 0.382 *, b* = 10.503 ± 0.348 *	37.057 ± 0.411 *	0.528 ± 0.307
	2	L* = 8.763 ± 0.358 *, a* = 36.273 ± 0.159 *, b* = 11.527 ± 0.429 *	38.062 ± 0.273 *	1.278 ± 0.121
	3	L* = 7.750 ± 0.213 *, a* = 35.007 ± 0.163 *, b* = 10.073 ± 0.165 *	36.427 ± 0.202 *	1.165 ± 0.358
	4	L* = 8.467 ± 0.145 *, a* = 35.557 ± 0.577 *, b* = 10.843 ± 0.501 *	37.176 ± 0.527 *	0.726 ± 0.157
	5	L* = 8.697 ± 0.308 *, a* = 36.220 ± 0.288 *, b* = 9.723 ± 0.250 *	37.503 ± 0.338 *	1.449 ± 0.210
	6	L* = 9.427 ± 0.012 *, a* = 33.490 ± 0.121 *, b* = 8.980 ± 0.135 *	34.673 ± 0.130 *	2.779 ± 0.056
	7	L* = 9.427 ± 0.04 *, a* = 33.473 ± 0.160 *, b* = 8.733 ± 0.182 *	34.594 ± 0.200 *	2.954 ± 0.188
	8	L* = 8.337 ± 0.038 *, a* = 34.227 ± 0.237 *, b* = 8.900 ± 0.148 *	35.365 ± 0.267 *	2.256 ± 0.070
	9	L* = 9.470 ± 0.078 *, a* = 34.417 ± 0.132 *, b* = 10.343 ± 0.058 *	35.937 ± 0.142 *	1.427 ± 0.198
	10	L* = 5.763 ± 0.006 *, a* = 31.213 ± 0.042 *, b* = 8.690 ± 0.121 *	32.401 ± 0.067 *	5.441 ± 0.204
	11	L* = 6.003 ± 0.318 *, a* = 31.970 ± 0.818 *, b* = 9.487 ± 0.586 *	33.349 ± 0.952 *	4.470 ± 0.796
	12	L* = 8.790 ± 0.130 *, a* = 34.340 ± 0.893 *, b* = 9.870 ± 0.098 *	35.731 ± 0.852 *	1.488 ± 0.055

Where C*: Chromaticity; TCD: Total color difference. Freshly harvested fruit juice with pulp (JC); juice extracted from intact fruit stored at 4 °C (J1); vacuum-packaged juice with pulp stored at 4 °C (J2); vacuum-packaged juice with pulp stored at −20 °C (J3); and vacuum-packaged, pasteurized juice with pulp stored at −20 °C (J4). Statistical analysis was performed using one-way ANOVA followed by Dunnett’s test and Student’s *t*-test, as described by Zar et al. [27] and Saneii and Doosti. [28]. Values sharing the same letter as the control (JC) show no significant difference, whereas values marked with an asterisk indicate statistically significant differences (*p* < 0.05).

## Data Availability

The original contributions presented in the study are included in the article, further inquiries can be directed to the corresponding authors.

## References

[1] Santos Díaz M.D.S., Barba de la Rosa A.-P., Héliès-Toussaint C., Guéraud F., Nègre-Salvayre A. (2017). *Opuntia* Spp.: Characterization and benefits in chronic diseases. Oxid. Med. Cell. Longev..

[2] Abdulkadir N., Solomon W.K., Woldetsadik K. (2022). Optimization of heat treatment and pH of red and white pear cactus [*Opuntiaficus-indica* (L.) mill.] fruit juice using response surface methodology. Food Res..

[3] Ferreira R.M., Costa A.M., Pinto C.A., Silva A.M.S., Saraiva J.A., Cardoso S.M. (2023). Impact of fermentation and pasteurization on the physico-chemical and phytochemical composition of *Opuntia ficus-indica* juices. Foods.

[4] Servicio de Información Agroalimentaria y Pesquera (SIAP) Panorama Agroalimentario 2019. Secretaria de Agricultura y Desarrollo Rural 2019. https://www.gob.mx/agricultura.

[5] Sumaya-Martínez M.T., Diéguez T.S., García E.A., Sampedro J.G. (2010). Innovacion de productos de alto valor agregado a partir de la tuna mexicana. Rev. Mex. Agronegocios.

[6] Ferreira R.M., Amaral R.A., Silva A.M.S., Cardoso S.M., Saraiva J.A. (2022). Effect of high-pressure and thermal pasteurization on microbial and physico-chemical properties of *Opuntia ficus-indica* juices. Beverages.

[7] Verón H.E., Gauffin Cano P., Fabersani E., Sanz Y., Isla M.I., Fernández Espinar M.T., Gil Ponce J.V., Torres S. (2019). Cactus pear (*Opuntia ficus-indica*) juice fermented with autochthonous *Lactobacillus plantarum* S-811. Food Funct..

[8] Aghajanzadeh S., Ziaiifar A.M., Verkerk R. (2023). Effect of thermal and non-thermal treatments on the color of citrus juice: A Review. Food Rev. Int..

[9] Pinela J., Ferreira I.C.F.R. (2017). Nonthermal physical technologies to decontaminate and extend the shelf-life of fruits and vegetables: Trends aiming at quality and safety. Crit. Rev. Food Sci. Nutr..

[10] Deepa G.T., Chetti M.B., Khetagoudar M.C., Adavirao G.M. (2013). Influence of vacuum packaging on seed quality and mineral contents in chilli (*Capsicum annuum* L.). J. Food Sci. Technol..

[11] Zhang S.J., Hu T.T., Liu H.K., Chen Y.Y., Pang X., Zheng L., Chang S., Kang Y. (2018). Moderate vacuum packing and low temperature effects on qualities of harvested mung bean (*Vigna radiata* L.) Sprouts. Postharvest Biol. Technol..

[12] Horwitz W., Latimer G.W., International A. (2005). Official Methods of Analysis of AOAC International.

[13] Albalasmeh A.A., Berhe A.A., Ghezzehei T.A. (2013). A New Method for rapid determination of carbohydrate and total carbon concentrations using UV spectrophotometry. Carbohydr. Polym..

[14] Czabaj S., Kawa-Rygielska J., Kucharska A., Kliks J. (2017). Effects of mead wort heat treatment on the mead fermentation process and antioxidant activity. Molecules.

[15] Moßhammer M.R., Stintzing F.C., Carle R. (2005). Colour studies on fruit juice blends from *Opuntia* and *Hylocereus cacti* and betalain-containing model solutions derived therefrom. Food Res. Int..

[16] Brand-Williams W., Cuvelier M.E., Berset C. (1995). Use of a free radical method to evaluate antioxidant activity. LWT-Food Sci. Technol..

[17] Benzie I.F.F., Strain J.J. (1996). The ferric reducing ability of plasma (FRAP) as a measure of “antioxidant power”: The FRAP assay. Anal. Biochem..

[18] Secretaría de Salud. Norma Oficial Mexicana (1995). Método para la Cuenta de Microorganismos Coliformes Totales en Placa. Diario Oficial de La Federación.

[19] Secretaría de Salud. Norma Oficial Mexicana (1995). Método para la Cuenta de Bacterias Aerobias en Placa. Diario Oficial de La Federación.

[20] Secretaría de Salud. Norma Oficial Mexicana (1995). Método para la Cuenta de Mohos y Levaduras en Alimentos. Diario Oficial de La Federación.

[21] Espinosa Manfugás J. (2020). Evaluación Sensorial de los Alimentos.

[22] Bolaños E.N.A., Moctezuma Y.C., Servia J.L.C., Gerónimo R.I.G., Hernández E.R.S., Guzmán I.V. (2012). Caracterización fisicoquímica de siete variedades de tuna (*Opuntia* spp.) color rojo-violeta y estabilidad del pigmento de las dos variedades con mayor concentración. Rev. IC.

[23] Felker P., Rodriguez S.D.C., Casoliba R.M., Filippini R., Medina D., Zapata R. (2005). Comparison of *Opuntia ficus indica* varieties of mexican and argentine origin for fruit yield and quality in Argentina. J. Arid. Environ..

[24] Karababa E., Coşkuner Y., Aksay S. (2004). Some physical fruit properties of cactus pear (*Opuntia* spp.) That grow wild in the eastern mediterranean region of Turkey. J. Prof. Assoc. Cactus.

[25] Aparicio-Fernández X., Loza-Cornejo S., Torres-Bernal M.G., Velázquez-Placencia N.J., Arreola-Nava H.J. (2017). Physicochemical characteristics of fruits from wild *Opuntia* species from two semiarid regions of Jalisco, Mexico. Polibotanica.

[26] Piga A. (2004). Cactus pear: A fruit of nutraceutical and functional importance. J. PACD.

[27] Zar J.H. (2010). Biostatistical Analysis.

[28] Saneii S.H., Doosti H. (2024). Practical Biostatistics for Medical and Health Sciences.

[29] Rodrigues C., Polesca C., Bicalho I., Souza V.G.L., Coelhoso I., Fernando A.L. (2025). Quality preservation and shelf-life extension of prickly pear (*Opuntia ficus-indica* L. mill) using edible coatings. Foods.

[30] Sato K., Izumi H. (2023). Viability of sublethally injured indicator and pathogenic coliform bacteria on fresh-cut cabbage during storage in an active MAP of 10% CO_2_. J. Microorg. Control.

[31] Khan M.I., Giridhar P. (2014). Enhanced chemical stability, chromatic properties and regeneration of betalains in *Rivina humilis* L. berry juice. LWT-Food Sci. Technol..

[32] Diniz-Mendes L., Bernardes E., De Araujo P.S., Panek A.D., Paschoalin V.M.F. (1999). Preservation of frozen yeast cells by trehalose. Biotechnol. Bioeng..

[33] Leong S.Y., Oey I. (2012). Effects of processing on anthocyanins, carotenoids and vitamin c in summer fruits and vegetables. Food Chem..

[34] Mullen W., Stewart A.J., Lean M.E.J., Gardner P., Duthie G.G., Crozier A. (2002). Effect of freezing and storage on the phenolics, ellagitannins, flavonoids, and antioxidant capacity of red raspberries. J. Agric. Food Chem..

[35] Neri L., Faieta M., Di Mattia C., Sacchetti G., Mastrocola D., Pittia P. (2020). Antioxidant activity in frozen plant foods: Effect of cryoprotectants, freezing process and frozen storage. Foods.

[36] Balali G.I., Yar D.D., Afua Dela V.G., Adjei-Kusi P. (2020). Microbial contamination, an increasing threat to the consumption of fresh fruits and vegetables in today’s world. Int. J. Microbiol..

[37] Polak N., Kalisz S., Hać-Szymańczuk E., Kruszewski B. (2024). Impact of conventional pasteurization, high temperature short time, ultra-high temperature, and storage time on physicochemical characteristics, bioactive compounds, antioxidant activity, and microbiological quality of fruit nectars. Foods.

[38] Chen G.-L., Zheng F.-J., Lin B., Lao S.-B., He J., Huang Z., Zeng Y., Sun J., Verma K.K. (2020). Phenolic and volatile compounds in the production of sugarcane vinegar. ACS Omega.

[39] Kesavan R.K., Gogoi S., Nayak P.K. (2023). Influence of thermosonication and pasteurization on the quality attributes of kutkura (*Meyna spinosa*) juice. Appl. Food Res..

[40] Shourove J.H., Zzaman W., Chowdhury R.S., Hoque M.D.M. (2020). Effect of thermal treatment on physicochemical stability and antioxidant properties of locally available underutilized star fruit juice. Asian Food Sci. J..

[41] Maskat M.Y., Tan S.M. (2011). Effect of heat treatment on the physico-chemical properties of mengkudu (*Morinda citrifolia*) extract. Int. Food Res. J..

[42] Igual M., García-Martínez E., Camacho M.M., Martínez-Navarrete N. (2010). Effect of thermal treatment and storage on the stability of organic acids and the functional value of grapefruit juice. Food Chem..

[43] Juhart J., Medic A., Jakopic J., Veberic R., Hudina M., Stampar F. (2023). Use of HPLC-MS to determine the loss of metabolites in apple juices under different storage conditions. Foods.

[44] Martínez-Flores H.E., Garnica-Romo M.G., Bermúdez-Aguirre D., Pokhrel P.R., Barbosa-Cánovas G.V. (2015). Physico-chemical parameters, bioactive compounds and microbial quality of thermo-sonicated carrot juice during storage. Food Chem..

[45] Tian Y., Sun L., Yang Y., Gou X., Niu P., Guo Y. (2018). Changes in the physicochemical properties, aromas and polyphenols of not from concentrate (NFC) Apple juice during production. CyTA-J. Food.

[46] Monroy-Gutiérrez T., Martínez-Damián M.T., Barrientos-Priego A.F., Gallegos-Vázquez C., Rodríguez-Pérez E. (2017). Evaluación de algunas características físicas y químicas de frutos de xocotuna, tuna y xoconostle en poscosecha. Rev. Mex. Cienc. Agríc.

[47] Ramoba L., Monyama M.C., Moganedi K. (2022). Storage potential of the cactus pear (*Opuntia ficus-indica*) fruit juice and its biological and chemical evaluation during fermentation into cactus pear wine. Beverages.

[48] El Kharrassi Y., Mazri M.A., Benyahia H., Benaouda H., Nasser B., El Mzouri E.H. (2016). Fruit and juice characteristics of 30 accessions of two cactus pear species (*Opuntia ficus indica* and *Opuntia megacantha*) from different regions of morocco. Lebensm.-Wiss. Technol..

[49] Padureanu C., Badarau C.L., Maier A., Padureanu V., Lupu M.I., Canja C.M., Branescu G.R., Bujor O.-C., Matei F., Poiana M.-A. (2023). Ultrasound treatment influence on antioxidant properties of blueberry vinegar. Fermentation.

[50] Hmid I., Elothmani D., Hanine H., Oukabli A., Mehinagic E. (2017). Comparative study of phenolic compounds and their antioxidant attributes of eighteen pomegranate (*Punica granatum* L.) Cultivars Grown in Morocco. Arab. J. Chem..

[51] Burin V.M., Falcão L.D., Gonzaga L.V., Fett R., Rosier J.P., Bordignon-Luiz M.T. (2010). Colour, phenolic content and antioxidant activity of grape juice. Ciênc. Tecnol. Aliment..

[52] Ochoa-Velasco C.E., Guerrero-Beltrán J.Á. (2013). Efecto de la temperatura de almacenamiento sobre las características de calidad de tuna blanca villanueva (*Opuntia albicarpa*). Rev. Iberoam. Tecnol. Postcosecha.

[53] Stintzing F.C., Herbach K.M., Mosshammer M.R., Carle R., Yi W., Sellappan S., Akoh C.C., Bunch R., Felker P. (2005). Color, Betalain pattern, and antioxidant properties of cactus pear (*Opuntia* spp.) Clones. J. Agric. Food Chem..

[54] Tkacz K., Chmielewska J., Turkiewicz I.P., Nowicka P., Wojdyło A. (2020). Dynamics of changes in organic acids, sugars and phenolic compounds and antioxidant activity of sea buckthorn and sea buckthorn-apple juices during malolactic fermentation. Food Chem..

[55] Kwaw E., Ma Y., Tchabo W., Apaliya M.T., Wu M., Sackey A.S., Xiao L., Tahir H.E. (2018). Effect of lactobacillus strains on phenolic profile, color attributes and antioxidant activities of lactic-acid-fermented mulberry juice. Food Chem..

[56] Janiszewska-Turak E., Pobiega K., Rybak K., Synowiec A., Woźniak Ł., Trych U., Gniewosz M., Witrowa-Rajchert D. (2023). Changes in physical and chemical parameters of beetroot and carrot juices obtained by lactic fermentation. Appl. Sci..

[57] Zin M.M., Borda F., Márki E., Bánvölgyi S. (2021). Betalains, total polyphenols, and antioxidant contents in red beetroot peel (*Cylindra type*). Progress.

[58] Gliszczyńska-Świgło A., Szymusiak H., Malinowska P. (2006). Betanin, the main pigment of red beet: Molecular origin of its exceptionally high free radical-scavenging activity. Food Addit. Contam..

[59] Li S., Tao Y., Li D., Wen G., Zhou J., Manickam S., Han Y., Chai W.S. (2021). Fermentation of blueberry juices using autochthonous lactic acid bacteria isolated from fruit environment: Fermentation characteristics and evolution of phenolic profiles. Chemosphere.

[60] Kwak M.-K., Liu R., Kang S.-O. (2018). Antimicrobial activity of cyclic dipeptides produced by *Lactobacillus plantarum* LBP-K10 against multidrug-resistant bacteria, pathogenic fungi, and influenza a virus. Food Control.

[61] Ferreira I., de Sousa Melo D., Menezes A.G.T., Fonseca H.C., de Assis B.B.T., Ramos C.L., Magnani M., Dias D.R., Schwan R.F. (2022). Evaluation of potentially probiotic yeasts and *Lactiplantibacillus plantarum* in co-culture for the elaboration of a functional plant-based fermented beverage. Food Res. Int..

[62] Sawicki T., Wiczkowski W. (2018). The Effects of boiling and fermentation on betalain profiles and antioxidant capacities of red beetroot products. Food Chem..

[63] Sadowska-Bartosz I., Bartosz G. (2022). Evaluation of the antioxidant capacity of food products: Methods, applications and limitations. Processes.

[64] Grover Y., Negi P.S. (2023). Recent Developments in freezing of fruits and vegetables: Striving for controlled ice nucleation and crystallization with enhanced freezing rates. J. Food Sci..

[65] Imenšek N., Kristl J., Kraner Šumenjak T., Ivančič A. (2021). Antioxidant activity of elderberry fruits during maturation. Agriculture.

[66] Sumaya-Martínez M.T., Cruz-Jaime S., Madrigal-Santillán E., García-Paredes J.D., Cariño-Cortés R., Cruz-Cansino N., Valadez-Vega C., Martinez-Cardenas L., Alanís-García E. (2011). Betalain, acid ascorbic, phenolic contents and antioxidant properties of purple, red, yellow and white cactus pears. Int. J. Mol. Sci..

[67] Knez E., Kadac-Czapska K., Grembecka M. (2025). Evaluation of spectrophotometric methods for assessing antioxidant potential in plant food samples—A critical approach. Appl. Sci..

[68] Rumpf J., Burger R., Schulze M. (2023). Statistical evaluation of DPPH, ABTS, FRAP, and Folin-Ciocalteu assays to assess the antioxidant capacity of lignins. Int. J. Biol. Macromol..

[69] Trych U., Buniowska-Olejnik M., Marszałek K. (2022). Bioaccessibility of betalains in beetroot (*Beta vulgaris* L.) juice under different high-pressure techniques. Molecules.

[70] Yvonne R.-M., Socorro Josefina V.-R., Luis Alfonso M., Judith Esmeralda U.-S. (2025). Impact of the food matrix on the antioxidant and hypoglycemic effects of betalains from red prickly pear juice after in vitro digestion. Foods.

[71] Renard C.M.G.C., Maingonnat J.F. (2012). Thermal processing of fruits and fruit juices. Thermal Food Processing: New Technologies and Qualities Issues.

[72] Nayak P.K., Chandrasekar C.M., Gogoi S., Kesavan R.K. (2022). Impact of thermal and thermosonication treatments of amora (*Spondius pinnata*) juice and prediction of quality changes using artificial neural networks. Biosyst. Eng..

[73] Wang X., Wang P. (2023). Red beetroot juice fermented by water kefir grains: Physicochemical, antioxidant profile and anticancer activity. Eur. Food Res. Technol..

[74] Değirmencioğlu N., Gurbuz O., Şahan Y. (2016). The monitoring, via an in vitro digestion system, of the bioactive content of vegetable juice fermented with *Saccharomyces cerevisiae* and *Saccharomyces boulardii*. J. Food Process. Preserv..

[75] Maskan M. (2006). Effect of thermal processing on tristimulus colour changes of fruits. Stewart Postharvest Rev..

